# Supplementary material for: Targeting AASS alleviates neurotoxicity and improves mitochondrial function in astrocyte models for pyridoxine-dependent epilepsy
Source: Mol Ther Nucleic Acids. 2025 Sep 30;36(4):102728. doi: 10.1016/j.omtn.2025.102728 (PMC12554910; doi:10.1016/j.omtn.2025.102728)
Supplement: Document S1. Figures S1–S12 [file mmc1.pdf]

OMTN, Volume *36*

Supplemental information

Targeting AASS alleviates neurotoxicity and improves mitochondrial function in astrocyte models for pyridoxine-dependent epilepsy

Imke M.E. Schuurmans, Udo Engelke, Muna Abedrabbo, Sofía Puvogel, Rachel Mijdam, Gijs-Jan Scholten, Sara B. van Katwijk, Astrid Oudakker, Hilal H. Al-Shekaili, Dirk J. Lefeber, Blair R. Leavitt, Clara D.M. van Karnebeek, Nael Nadif Kasri, and Alejandro Garanto

Supplemental Figures

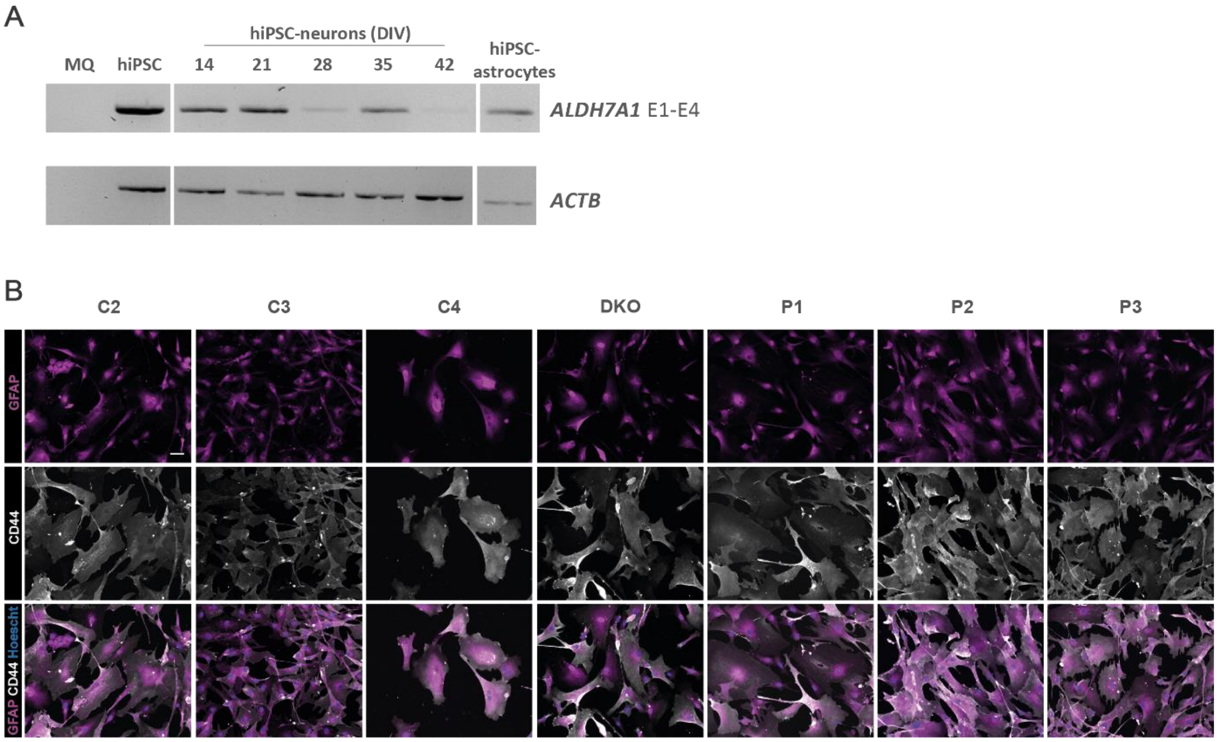

Figure S1. Characterization of all PDE astrocytes. (A). Expression of *ALDH7A1* relative to *ACTB* by regular PCR in hiPSCs, hiPSC-derived neurons from 14, 21, 28, 35 and 42 days in vitro (DIV) and hiPSC-derived astrocytes. (**B).** Representative images of immunostaining of GFAP (magenta), CD44 (white), GLUD1 (magenta) and ALDH1L1 (white) in DIV 35 astrocytes from C2, C3, C4, *ALDH7A1/AASS* KO, P1, P2 and P3. All pictures were taken at the same magnification (scale bar = 50 μm).

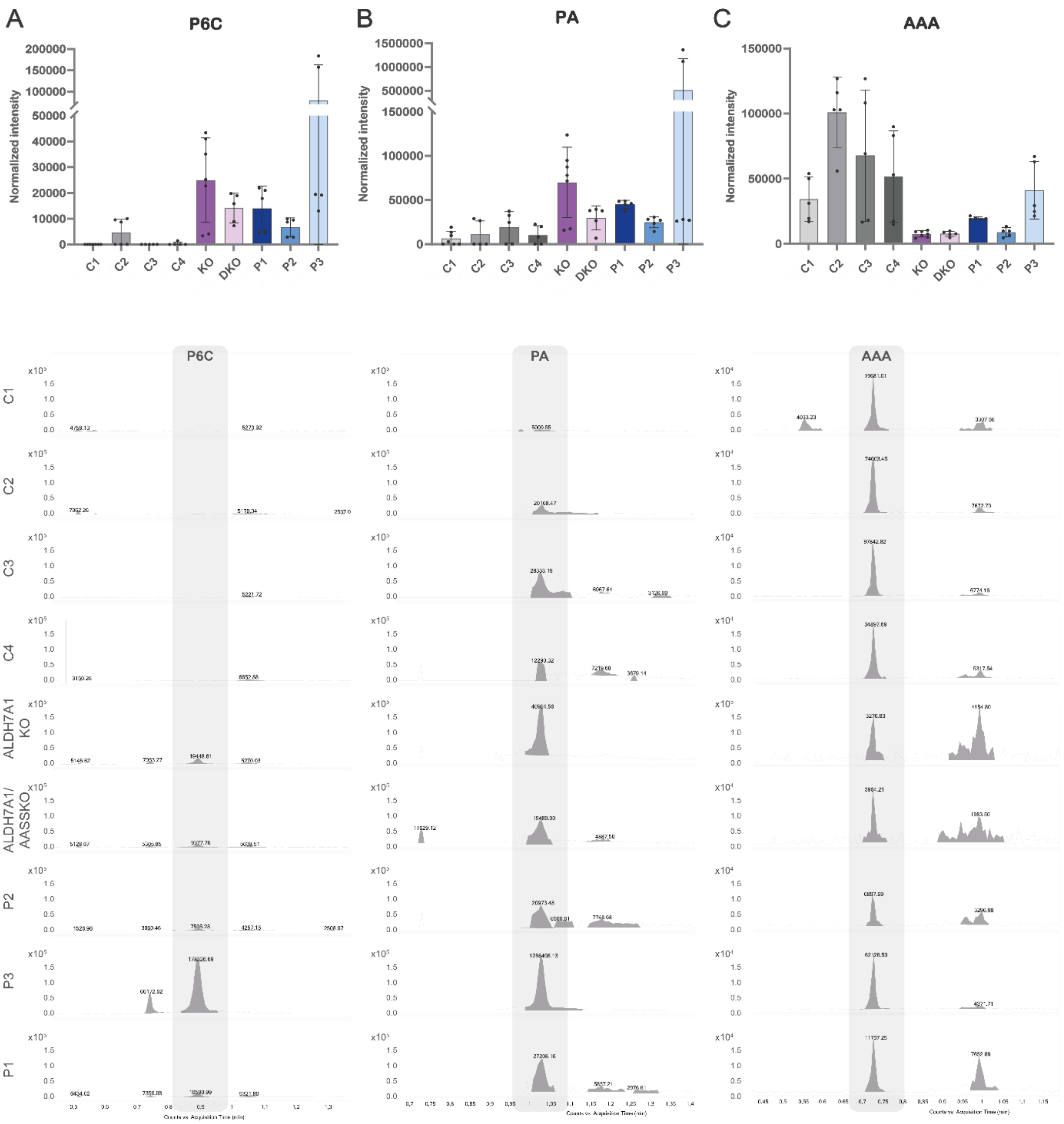

Figure S2. Metabolic characterization of all PDE astrocytes. (A). The graph shows the normalized intensity of P6C relative to phenylalanine measured via NGMS in astrocytes from control, *ALDH7A1* KO (KO), *ALDH7A1/AASS* DKO (DKO) and PDE patients. *n* = 5 for C1; *n* = 5 for C2; *n* = 5 for C3; *n* = 5 for C4; *n* = 6 for KO; *n* = 4 for DKO; *n* = 5 for P1; *n* = 5 for P2; *n* = 5 for P3. For one sample of the biological triplicate of each line the P6C peak at the correct retention time (indicated by grey box) is shown for all lines, including the relative peak intensity. **(B).** The graph shows the normalized intensity of PA relative to the housekeeping metabolite phenylalanine measured via NGMS in astrocytes from control, KO, DKO and PDE patients. *n* = 5 for C1; *n* = 5 for C2; *n* = 5 for C3; *n* = 5 for C4; *n* = 6 for KO; *n* = 4 for DKO; *n* = 5 for P1; *n* = 5 for P2; *n* = 5 for P3. For

one sample of the biological triplicate of each line the PA peak at the correct retention time (indicated by grey box) is shown for all lines, including the relative peak intensity. **(C).** The graph shows the normalized intensity of AAA relative to the housekeeping metabolite phenylalanine measured via NGMS in astrocytes from control, KO, DKO and PDE patients. *n* = 5 for C1; *n* = 5 for C2; *n* = 5 for C3; *n* = 5 for C4; *n* = 6 for KO; *n* = 4 for DKO; *n* = 5 for P1; *n* = 5 for P2; *n* = 5 for P3. For one sample of the biological triplicate of each line the AAA peak at the correct retention time (indicated by grey box) is shown for all lines, including the relative peak intensity.

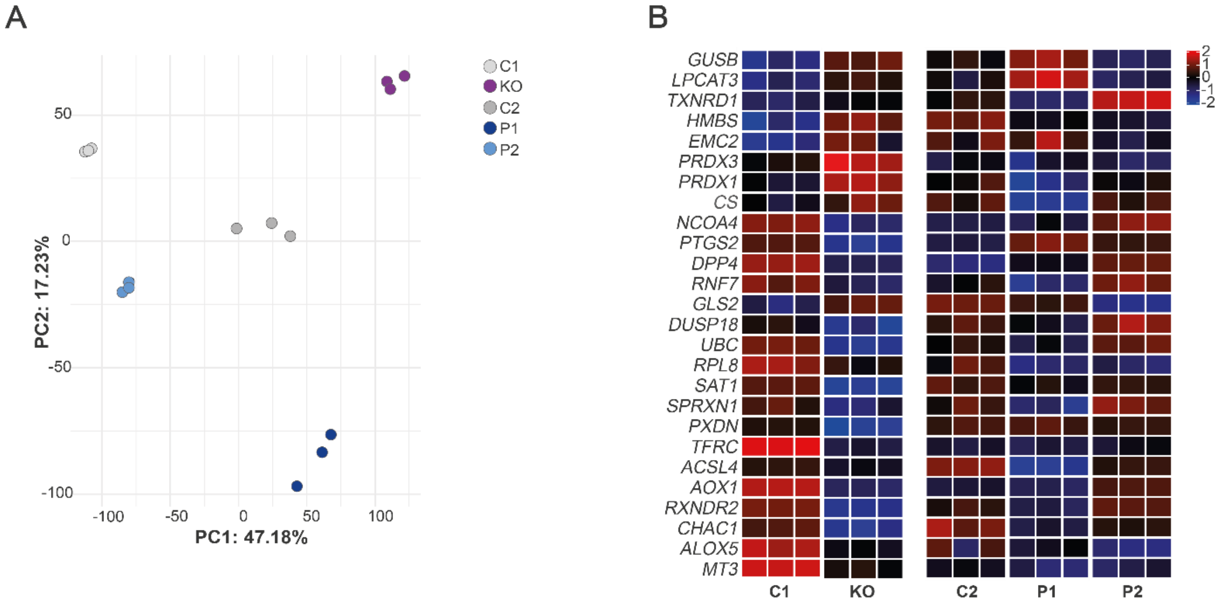

Figure S3. Principal component plot of RNA sequencing. (A) Principal component (PC) plot of RNA sequencing data representing biological triplicates of DIV 35 astrocytes derived from *ALDH7A1* KO (KO), P1, P2 and C1 and C2. **(B).** Heatmap of gene expression from the C1, KO, C2, P1 and P2 astrocytes of genes associated with lipid peroxidation related oxidative stress.

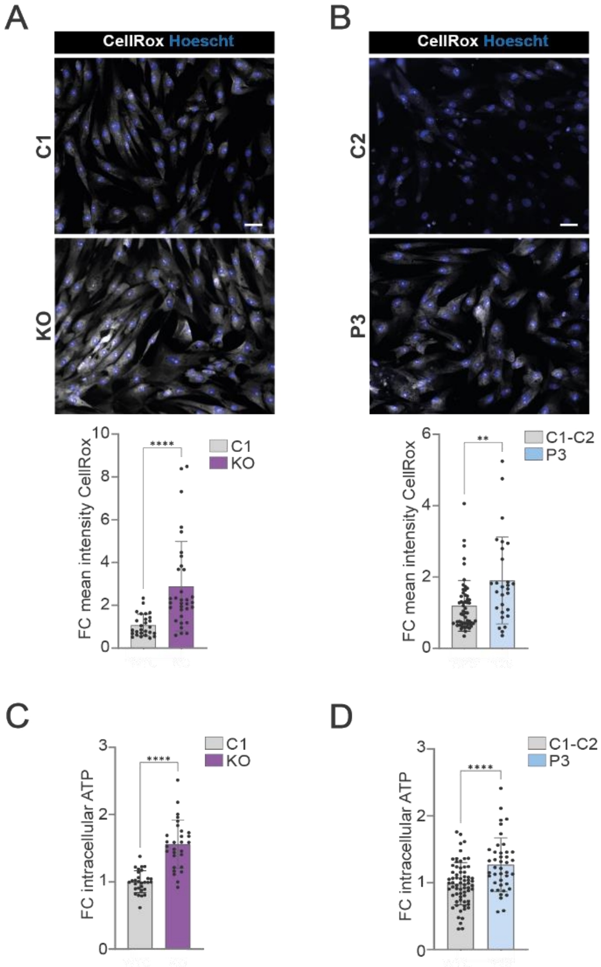

Figure S4. ROS and ATP levels in PDE astrocytes. FC of relative ATP concentration through ATP determination kit in astrocytes derived from *ALDH7A1* KO (KO) versus C1 **(A)** and in PDE P3 (P3) versus control (C1-C2) **(B)**. *n* = 29/3 for C1; *n* = 29/3 for KO; *n* = 67/3 for C1-C2 and *n* = 42/3 for P3. Statistically significant differences were tested through unpaired t-test. Representative images of CellRox assay (Scale bar = 50 µm) and FC of mean intensity of CellRox per well relative to average intensity of control shown for *ALDH7A1* KO (KO) versus C1 **(C)** and in PDE P3 (P3) versus control (C1-C2) **(D)**. *n* = 27/4 for C1; *n* = 33/4 for KO; *n* = 55/4 for C1-C2 and *n* = 28/4 for P3. Statistically significant differences were tested through ordinary one-way ANOVA and Dunnett's multiple comparison test. EF.

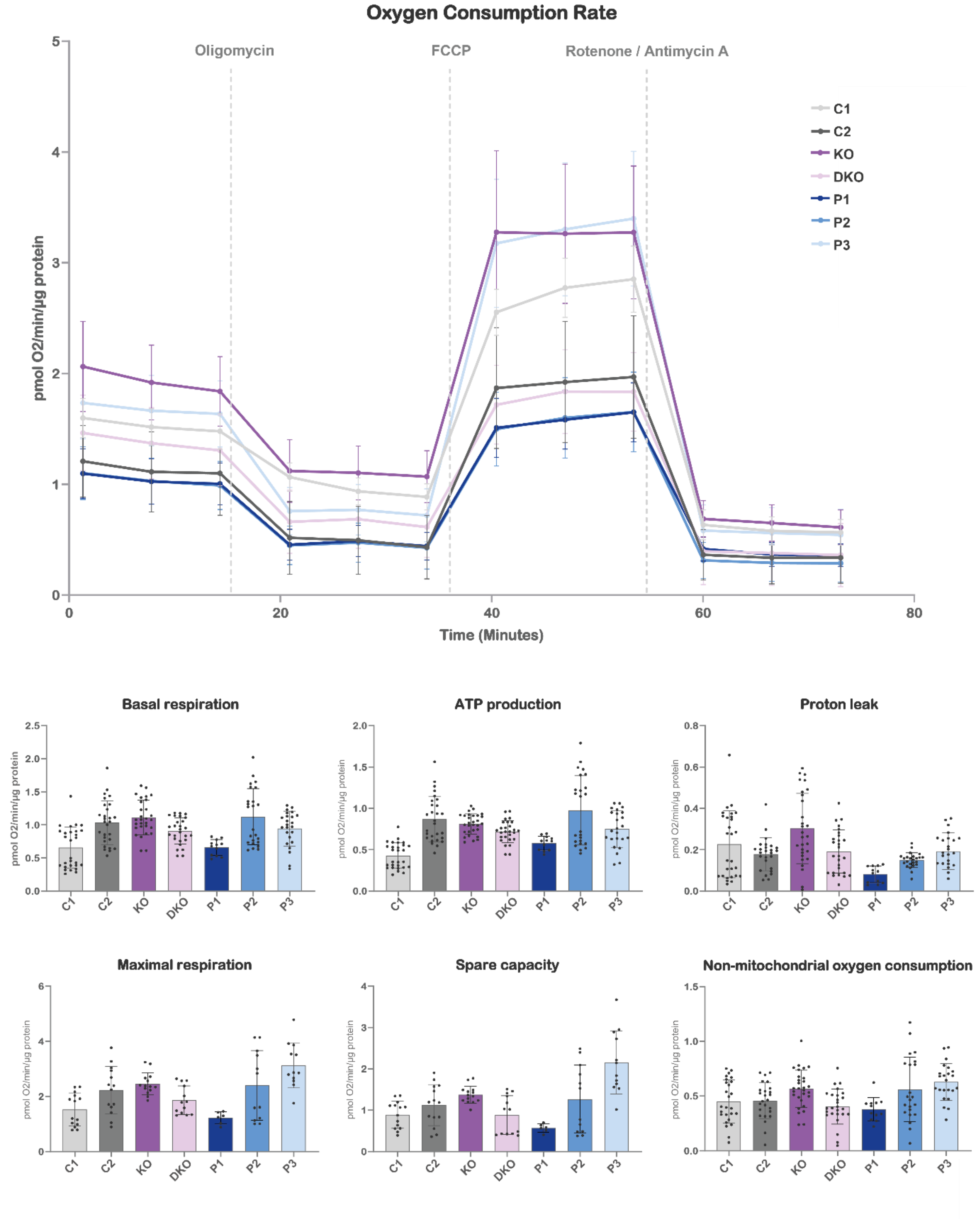

Figure S5. Seahorse assay. OCR plot as well as Basal respiration (BR), ATP production (AP), proton leak (PL), maximal respiration (MR), spare capacity (SC) and non-mitochondrial oxygen consumption (NMOC) represented in bar graphs are shown for DIV 35 astrocytes from C1, C2, *ALDH7A1* KO (KO), *ALDH7A1/AASS* DKO (DKO) and PDE patient lines. For BR, AP, PL and NMOC: *n*

= 28/2 for C1; *n* = 27/2 for C2; *n* = 29/2 for KO; *n* = 27/2 for DKO; *n* = 24/2 for P1; *n* = 24/2 for P2; *n* = 12/1 for P3. For MR and CP: *n* = 14/2 for C1; *n* = 14/2 for C2; *n* = 14/2 for KO; *n* = 13/2 for DKO; *n* = 12/2 for P1; *n* = 12/2 for P2; *n* = 6/1 for P3.

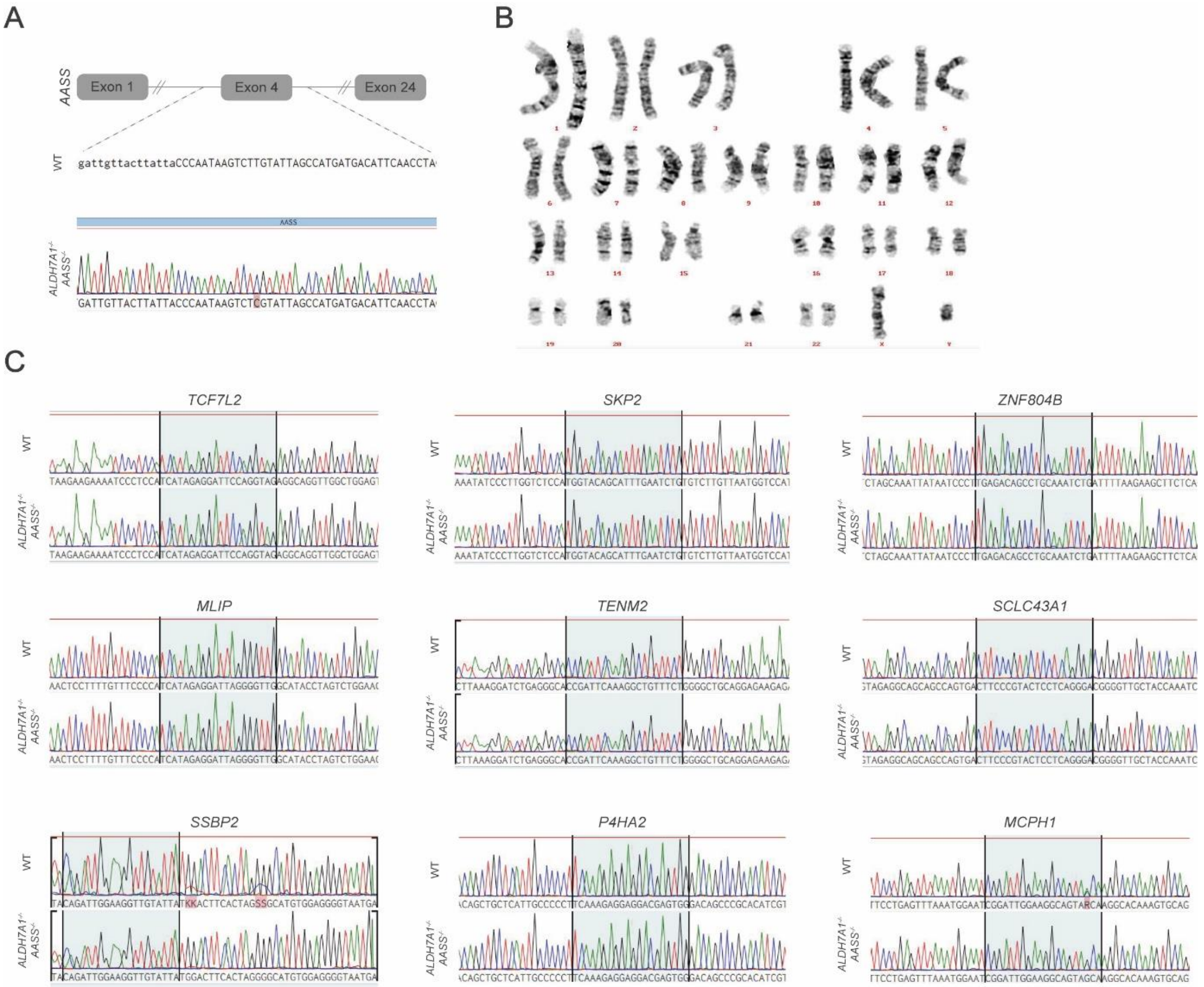

Figure S6. Generation of *ALDH7A1/AASS* DKO hiPSC line. (A). Schematic overview of CRISPR/Cas9 editing to create *ALDH7A1/AASS* DKO hiPSC line including chromatograms of sequencing results. **(B).** Normal karyotype of *ALDH7A1/AASS* DKO hiPSC. **(C).** All predicted off-target sites have been sequenced and no mutations were detected.

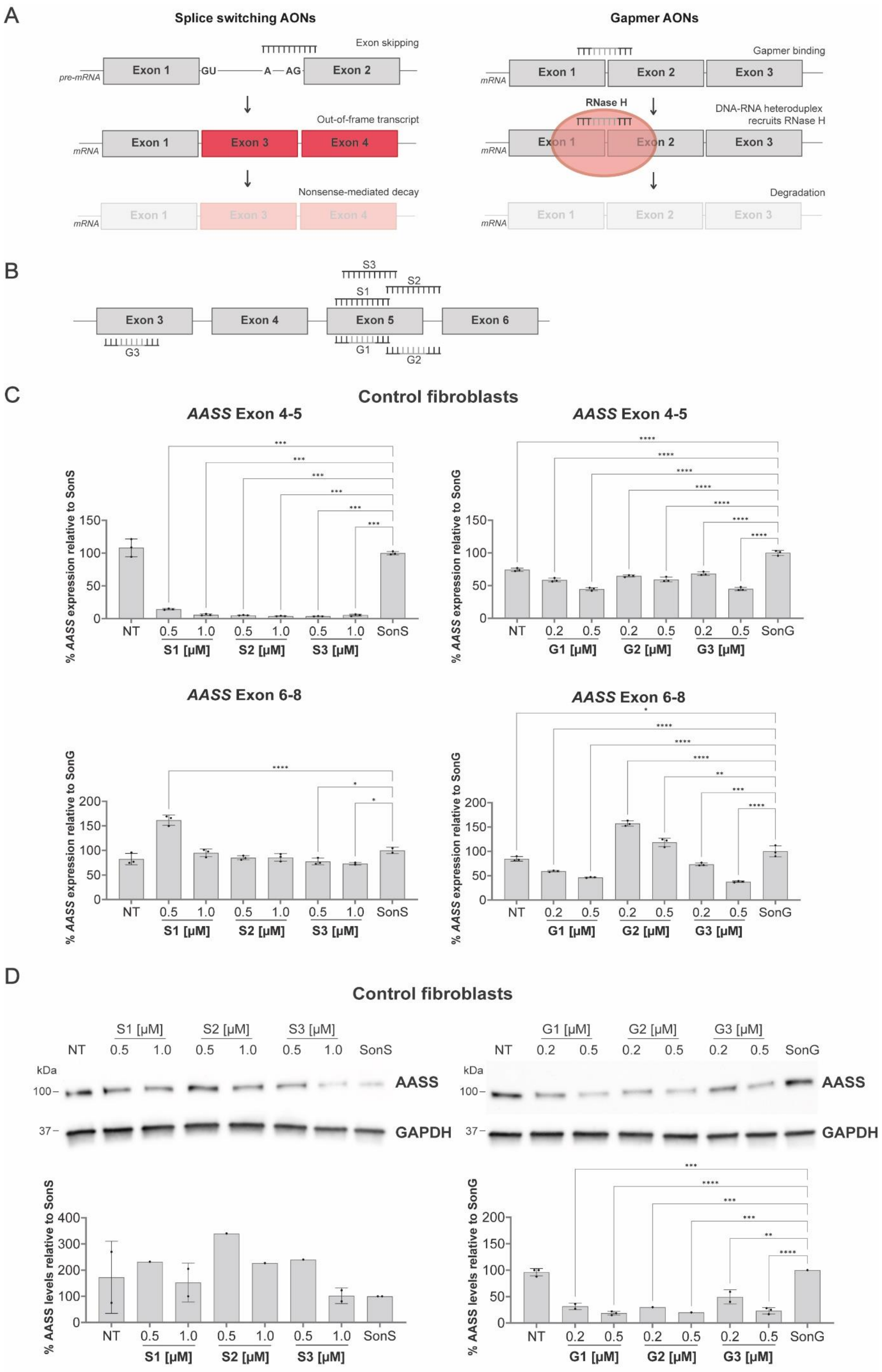

A
Splice switching AONs
Exon skipping
pre-mRNA
Exon 1
GU
A
AG
Exon 2
Out-of-frame transcript
mRNA
Exon 1
Exon 3
Exon 4
Nonsense-mediated decay
Gapmer AONs
Gapmer binding
mRNA
Exon 1
Exon 2
Exon 3
RNase H
DNA-RNA heteroduplex recruits RNase H
Degradation
B
S3
S2
S1
Exon 3
Exon 4
Exon 5
Exon 6
G3
G1
G2
C
Control fibroblasts
AASS Exon 4-5
% AASS expression relative to SonS
% AASS expression relative to SonG
NT
0.5
1.0
SonS
S1 [µM]
S2 [µM]
S3 [µM]
0.2
SonG
G1 [µM]
G2 [µM]
G3 [µM]
AASS Exon 6-8
D
Control fibroblasts
kDa
100
37
AASS
GAPDH
% AASS levels relative to SonS
% AASS levels relative to SonG

Figure S7. Screening of AONs targeting *AASS* in fibroblasts. (A). Schematic representation of two AON-strategies to target *AASS*; splice-switching AONs (ssAONs) and gapmers. **(B).** Overview of the three designed ssAONs (top) and three gapmers (bottom). **(C).** Relative expression of the regions spanning exon 4-5 and exon 6-8 of *AASS* normalized for *GUSB* by RT-qPCR in control fibroblasts four days upon AON-delivery. ssAONs were transfected at 0.5 and 1.0 µM, gapmers were transfected at 0.2 and 0.5 µM, SonS was transfected at 1.0 µM and SonG was transfected at 0.5 µM**.** Data represents the percentage of remaining *AASS* expression relative to the SonG condition. **(D).** Semi-quantification of AASS protein levels relative to GAPDH and representative (cropped) western blot of control fibroblasts four days upon AON-delivery. ssAONs were transfected at 0.5 and 1.0 µM, gapmers were transfected at 0.2 and 0.5 µM, SonS was transfected at 1.0 µM and SonG was transfected at 0.5 µM. Data represents the percentage of remaining AASS expression relative to the SonG condition. For exact *n* per experiment, per condition see Table S1.

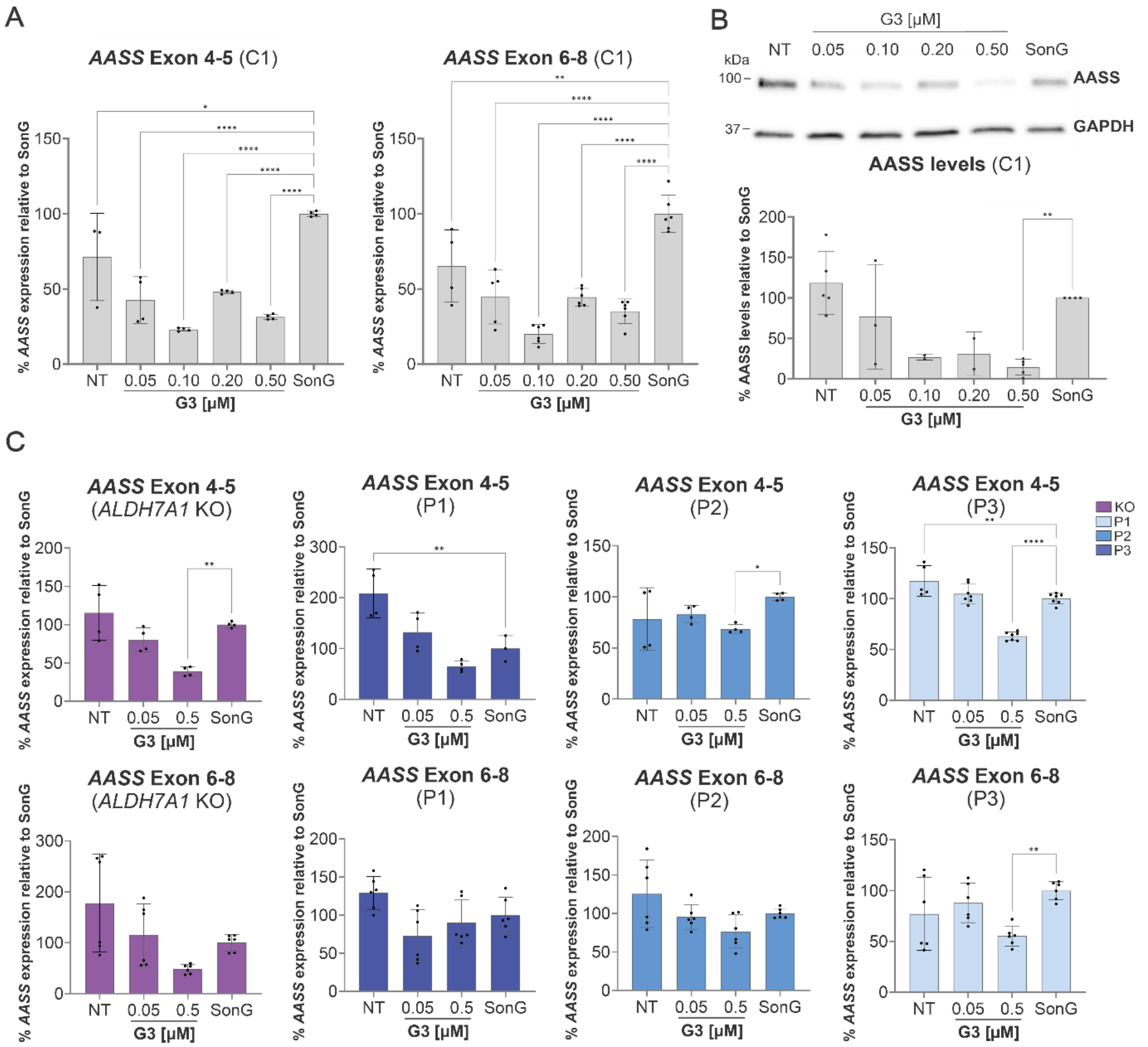

Figure S8. Screening of gapmers targeting *AASS* in control and PDE patient-derived astrocytes. (A). Relative expression of the regions exon 4-5 and exon 6-8 of *AASS* by qPCR in C1 astrocytes seven days upon G3-delivery at concentrations ranging from 0.05 - 0.5 µM and a sense oligonucleotide (SonG) control at 0.5 µM. Expression of genes was normalized against *GUSB*. Data represents the percentage of remaining *AASS* expression relative to the SonG condition. **(B).** Semi-quantification of AASS protein levels relative to GAPDH and representative (cropped) western blot of C1 astrocytes seven days upon delivery G3 at concentrations ranging from 0.05 - 0.5 µM and SonG at 0.5 µM. Data represents the percentage of remaining AASS levels relative to the SonG condition. **(C).** Relative expression of the region spanning exon 4-5 and exon 6-8 of *AASS* by qPCR in *ALDH7A1* KO, P1, P2 and P3 astrocytes seven days upon G3-delivery at 0.05 / 0.5 µM concentrations and SonG at 0.5 µM. Expression of genes was normalized against *GUSB*. Data represents the percentage of remaining *AASS* expression relative to the SonG condition. For exact *n* per experiment, per condition see Table S1.

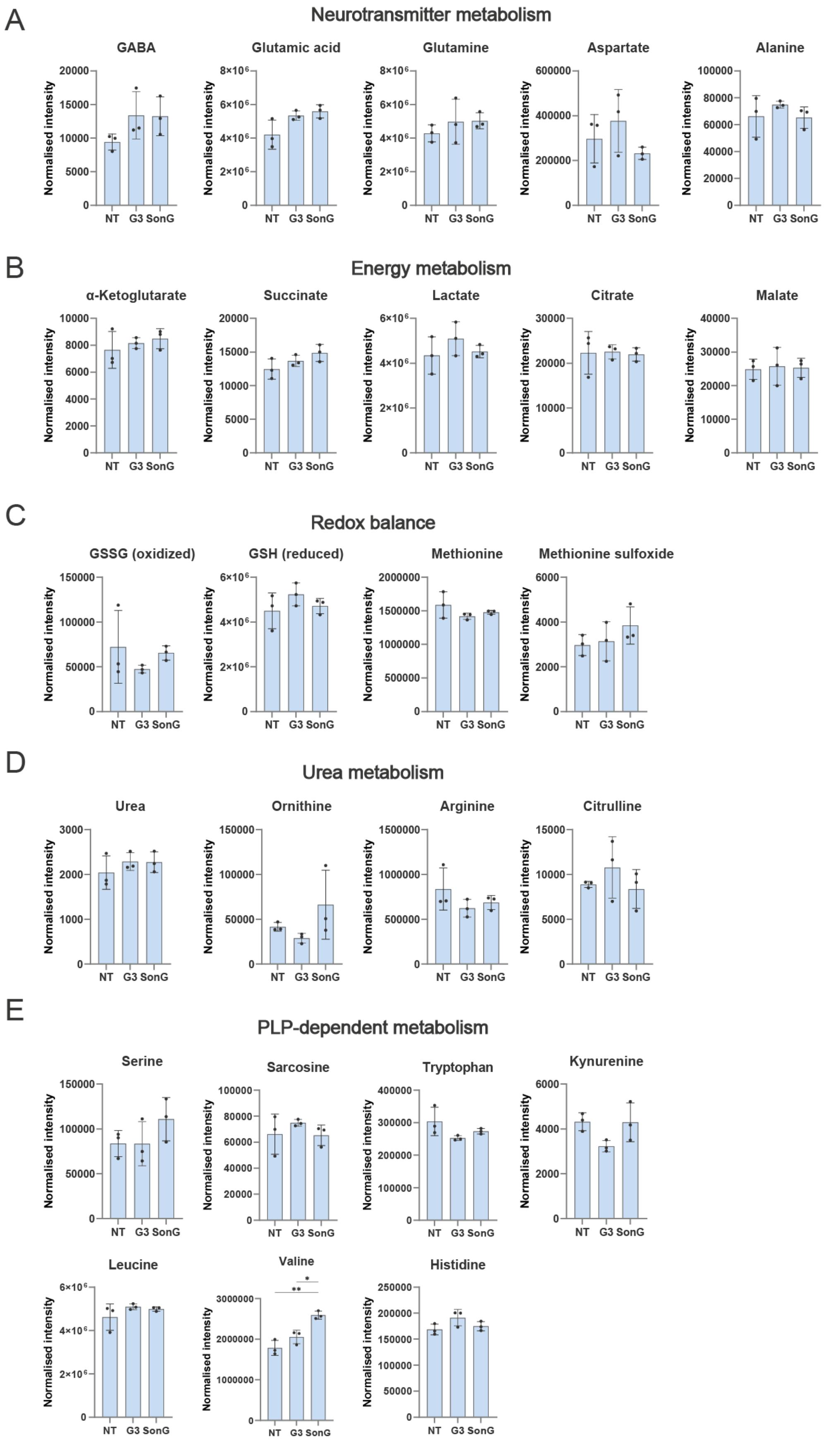
A
Neurotransmitter metabolism
GABA
Glutamic acid
Glutamine
Aspartate
Alanine
B
Energy metabolism
α-Ketoglutarate
Succinate
Lactate
Citrate
Malate
C
Redox balance
GSSG (oxidized)
GSH (reduced)
Methionine
Methionine sulfoxide
D
Urea metabolism
Urea
Ornithine
Arginine
Citrulline
E
PLP-dependent metabolism
Serine
Sarcosine
Tryptophan
Kynurenine
Leucine
Valine
Histidine
Normalised intensity
NT
G3
SonG
*
**

Figure S9. Metabolite analysis of pathways potentially affected by *AASS* knockdown. Metabolites were analyzed in PDE P3 astrocytes under non-treated (NT), G3 and a sense oligonucleotide (SonG) control at 0.5 µM. Metabolites were grouped according to functional pathways potentially affected by *AASS* downregulation: **(A)** neurotransmitter metabolism (GABA, glutamic acid, glutamine, aspartate, alanine), **(B)** energy metabolism (α-ketoglutarate, succinate, lactate, citrate, malate), **(C)** redox balance (oxidized glutathione (GSSG), reduced glutathione (GSH), methionine, methionine sulfoxide), **(D)** urea metabolism (urea, ornithine, arginine, citrulline), and **(E)** PLP-dependent metabolism (serine, sarcosine, tryptophan, kynurenine, leucine, valine, histidine). Statistically significant differences were tested through ordinary one-way ANOVA and Dunnett's multiple comparison test.

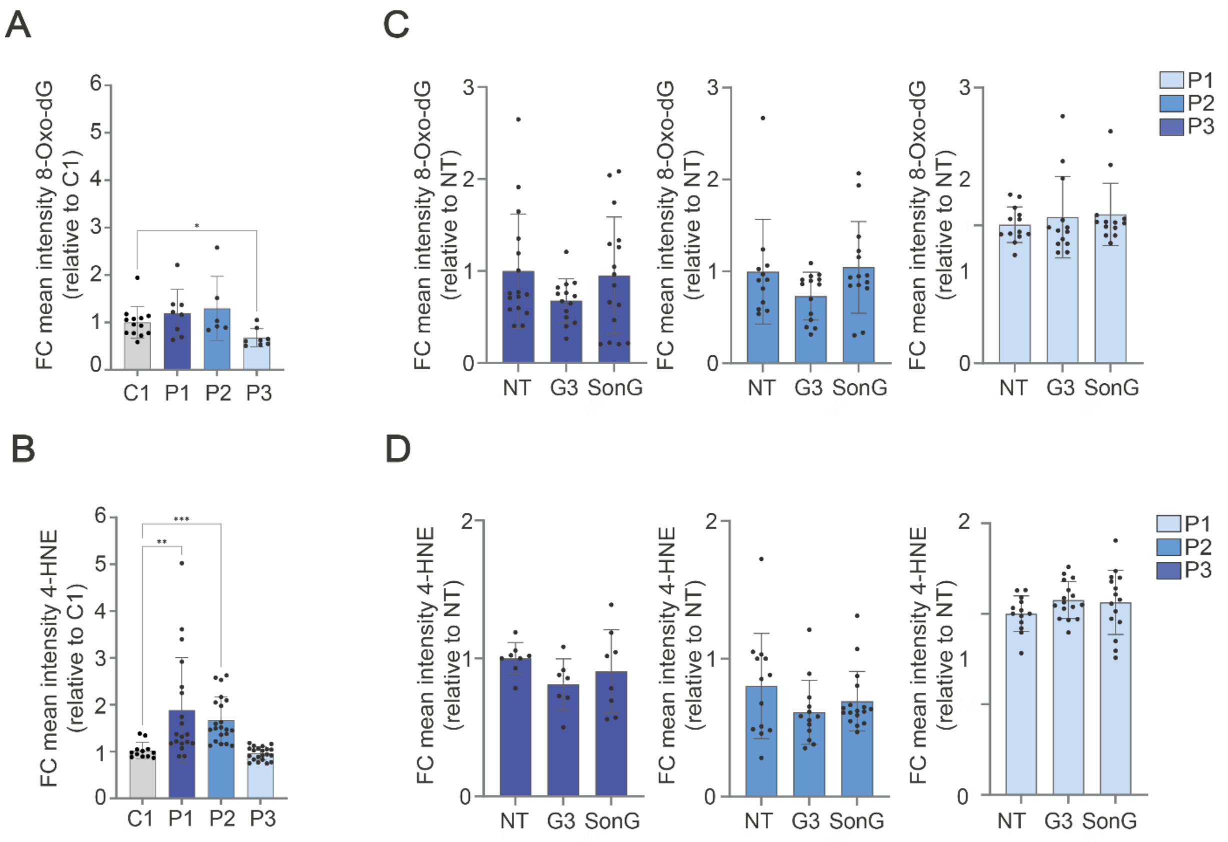

Figure S10. Oxidative stress upon gapmer treatment in PDE patient-derived astrocytes. (A). FC of mean intensity of 8-Oxo-dG per well relative to average intensity of C1 shown for P1 NT, P2 NT and P3 NT. **(B).** FC of mean intensity of 4-HNE per well relative to average intensity of C1 shown for P1 NT, P2 NT and P3 NT. **(C).** FC of mean intensity of 8-Oxo-dG per well relative to average intensity of corresponding NT shown for NT, G3 and SonG conditions in P1, P2 and P3 astrocytes. **(D).** FC of mean intensity of 4-HNE per well relative to average intensity of corresponding NT shown for NT, G3 and SonG conditions in P1, P2 and P3 astrocytes. For exact *n* per experiment, per condition see Table S1.

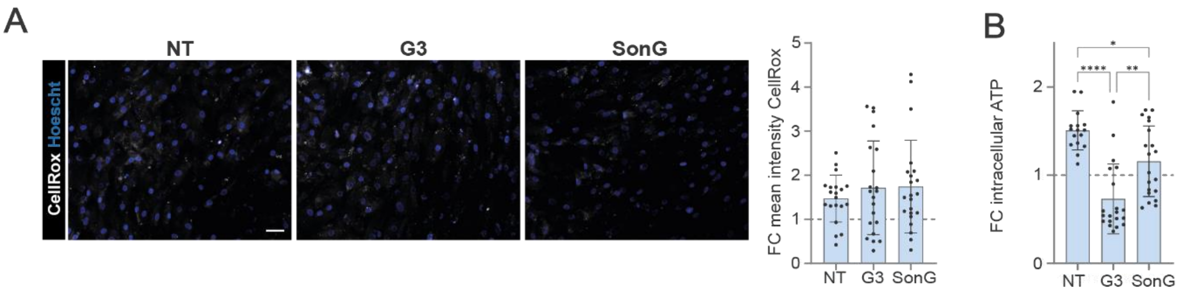

Figure S11. ROS and ATP levels in PDE P3 astrocytes upon gapmer treatment. Both experiments included non-treated control (NT), G3 at 0.5 µM and a sense oligonucleotide (SonG) control at 0.5 µM. **(A)** Fold Change (FC) of ATP concentration through ATP determination kit of P3 astrocytes relative to NT C1 under NT, G3, and SonG conditions. *n* = 16/3 for NT; *n* = 20/3 for G3 and *n* = 20/3 for SonG. Statistically significant differences were tested through ordinary one-way ANOVA with Dunnett's multiple comparison test. **(B)** Representative images of CellRox assay (Scale bar = 50 µm) and FC of mean intensity of CellRox of P3 astrocytes per well relative to average intensity of NT control under NT, G3, and SonG conditions. *n* = 20/3 for NT; *n* = 21/3 for G3 and *n* = 22/3 for SonG. Statistically significant differences were tested through ordinary one-way ANOVA and Dunnett's multiple comparison test.

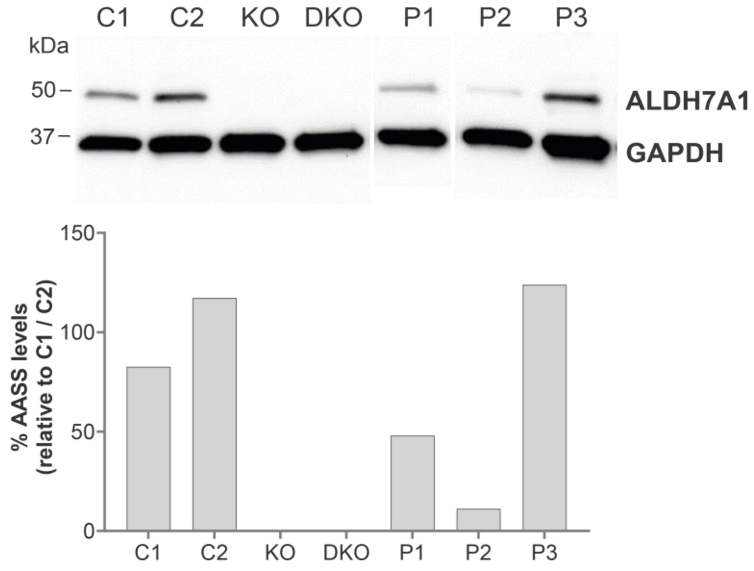

Figure S12. ALDH7A1 expression in the different astrocyte lines. Semi-quantification of ALDH7A1 protein levels relative to GAPDH and (cropped) western blot of C1, C2, *ALDH7A1* KO (KO), *ALDH7A1/AASS* DKO (DKO), P1, P2 and P3 astrocytes. Data represents the percentage of remaining ALDH7A1 expression relative to the average of C1 and C2.

Table S1. List of primers, clinical data on PDE patients and statistical information.
